# Olfactory receptors contribute to progression of kidney fibrosis

**DOI:** 10.1038/s41540-022-00217-w

**Published:** 2022-02-18

**Authors:** Ali Motahharynia, Shiva Moein, Farnoush Kiyanpour, Kobra Moradzadeh, Moein Yaqubi, Yousof Gheisari

**Affiliations:** 1 Regenerative Medicine Research Center, Isfahan University of Medical Sciences, Isfahan, Iran; 2 Department of Neurology and Neurosurgery, Montreal Neurological Institute and Hospital, McGill University, Montreal, Canada; 3 Department of Genetics and Molecular Biology, Isfahan University of Medical Sciences, Isfahan, Iran

**Keywords:** Time series, Dynamic networks, Regulatory networks, Genetic interaction

## Abstract

Olfactory receptors (ORs) which are mainly known as odor-sensors in the olfactory epithelium are shown to be expressed in several non-sensory tissues. Despite the specified role of some of these receptors in normal physiology of the kidney, little is known about their potential effect in renal disorders. In this study, using the holistic view of systems biology, it was determined that ORs are significantly changed during the progression of kidney fibrosis. For further validation, common differentially expressed ORs resulted from reanalysis of two time-course microarray datasets were selected for experimental evaluation in a validated murine model of unilateral ureteral obstruction (UUO). Transcriptional analysis by real-time quantitative polymerase chain reaction demonstrated considerable changes in the expression pattern of *Olfr433, Olfr129, Olfr1393, Olfr161*, and *Olfr622* during the progression of kidney fibrosis. For localization of these ORs, single-cell RNA-sequencing datasets of normal and UUO mice were reanalyzed. Results showed that *Olfr433* is highly expressed in macrophages in day-2 and 7 post-injury in UUO mice and not in normal subgroups. Besides, like previous findings, *Olfr1393* was shown to be expressed prominently in the proximal tubular cells of the kidney. In conclusion, our combinatorial temporal approach to the underlying mechanisms of chronic kidney disease highlighted the potential role of ORs in progression of fibrosis. The expression of *Olfr433* in the macrophages provides some clue about its relation to molecular mechanisms promoted in the fibrotic kidney. The proposed ORs in this study could be the subject of further functional assessments in the future.

## Introduction

Olfactory receptors (ORs), belonging to a super-family of G protein-coupled receptors, are well-recognized for their role in odor-sensation in the olfactory epithelium^1^. This family of receptors was first discovered by Linda Buck and Richard Axel^1^, leading to a Nobel prize in 2004^2^. More investigations on ORs determined that they are not only expressed in the olfactory epithelium but also non-sensory organs^3^. The study by Parmentier et al. determined the functionality of these receptors in sperm chemotaxis during fertilization^4^. Furthermore, it was demonstrated that these receptors have roles in cytoskeletal remodeling, pulmonary hyperplasia^5^, angiogenesis^6^, as well as heart metabolism in the cardiovascular system^7^. Besides, identification of the role of these receptors in other parts of the body, such as skin^8,9^, gastrointestinal^10–12^, and the immune system^13,14^ highlights the importance of further investigations on their function in the body.

One of the tissues, in which the presence and function of ORs are investigated, is the kidney^15–19^. Studies using unsupervised high-throughput techniques have discovered the existence of these receptors in the kidney^20,21^. Nevertheless, few studies have specifically focused on their role in renal function. The study by Pluznick et al. determined *Olfr78* role in blood pressure regulation through interactions with the byproducts of gut microbiota^22^. Furthermore, Shepard et al. demonstrated that *Olfr1393* participates in glucose transportation in both normal^23^ and pathological^24^ states of the kidney. Considering the magnitude of this gene family (around 1000 genes in the mouse and 400 genes in the human), as well as their role in chemosensation^19,25^, more investigations are needed to uncover the role of other OR subtypes in kidney function and hemostasis. Despite the above-mentioned few findings on the role of these receptors in the normal state of the kidney, no remarkable study has focused on their function in the progression of kidney fibrosis. Renal fibrosis is the common pathological manifestation of a variety of disorders leading to chronic kidney disease (CKD)^26,27^ which causes vast tubular atrophy and glomerulosclerosis^27^. Unfortunately, dialysis and kidney transplantation are the only treatments in the progressive states of the disease which cause serious complications^27^. In this regard, basic studies on the molecular mechanisms of the disease are crucial for developing new treatment strategies.

In this study, two time-course microarray datasets from a mouse model of unilateral ureteral obstruction (UUO) were reanalyzed and the gene interaction networks of differentially expressed genes (DEGs) were constructed. Experimental evaluation of common ORs between two datasets determined significant changes in the expression patterns of these genes during the progression of kidney fibrosis. Moreover, by analyzing single-cell RNA-sequencing (scRNA-seq) datasets, we could find some clues about the localization and function of these ORs.

## Results

In order to investigate the underlying molecular mechanisms activated during the progression of CKD, two time-course microarray datasets, GSE36496^28^ and GSE96571^29^ were reanalyzed. GSE36496 dataset contains transcriptomics data of the UUO and sham-operated C57BL/6 mice at days-1, 2, 5, and 9 postoperation^28^. The results of this study by Wu et al. suggest CEBPB and HNF4A signaling pathways as important regulators of kidney fibrosis^28^. The other dataset, GSE96571 which is also deposited by Wu et al., comprises the UUO and sham samples at hours-0.5, 1, 3, 5, 7, 12, as well as days-1, 3, 5, and 7 postoperation^29^. The single-time point analysis of this dataset by Wu et al. determined overexpression of stress responder genes in the first hours of obstruction besides nephrotoxic damage-related genes at later time points^29^. This dataset was used for the validation of our findings from the first dataset.

### Unsupervised evaluation of microarray dataset determined the quality of datasets

**Fig. 1:**
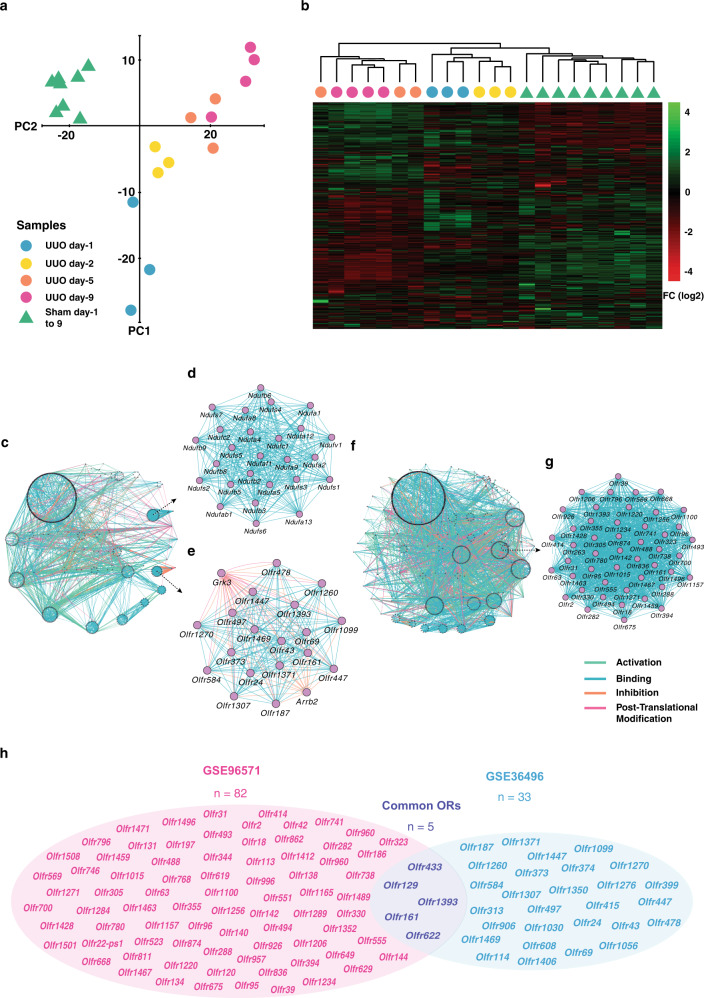
Network analysis of two microarray datasets on a mouse model of ureteral obstruction. Principal component analysis (PCA) and heatmap clustering of GSE36496 dataset demonstrated good quality of samples regarding treatment and times of harvest (**a, b**). Gene interaction network created from 2583 differentially expressed genes (DEGs) of GSE36496 dataset (**c**). The first- (**d**) and the second-ranked (**e**) modules resulted from topological analysis of the network constitute NDUF and olfactory receptor (OR) gene families, respectively. Gene Interaction network from 3176 DEGs of GSE96571 dataset (**f**). The top-ranked module from topological analysis of the constructed network is constituted of ORs (**g**). The overlap between differentially expressed ORs of GSE36496 and GSE96571 datasets (**h**).

The quality assessment of the GSE36496 dataset by principal component analysis (PCA) demonstrated the separation of sham and UUO groups from each other. Furthermore, the UUO samples were separated based on the times of harvest, which determines the model quality (Fig. 1a). This result was also validated by the hierarchical clustering (Fig. 1b). The DEGs were determined using the linear models for microarray data (LIMMA)^30^ Package of R software^31^, which according to our recent study^32^ is the most reliable tool for time-course analysis of microarray data. A Comparison of the UUO and sham samples determined 2583 DEGs with an adjusted *p* value < 0.05. The DEGs were used for the construction of a gene interaction network and further topological analysis (Fig. 1c).

### ORs constitute a dense module in the interactome map of kidney fibrosis

In order to discover the functional units of the network, module analysis was performed. Modules are individual units of biological networks that are similar in physical, chemical, or functional aspects and supposed to have a specific function in the networks^33,34^. Assessment of densely connected network regions, based on the clustering coefficient, revealed 36 significant modules. The first and second modules were mainly related to NDUF and ORs signaling pathways (Fig. 1d, e). NADH:ubiquinone oxidoreductase supernumerary subunits (NDUF) family of genes are expressed in the mitochondria and their relation to kidney fibrosis has previously been demonstrated by Granata et al.^35^. Despite evidence on the contribution of *Olfr1393* to the progression of type 2 diabetes^24^, no study has shown the relation of ORs to kidney fibrosis. Two non-OR genes, *Arrb2* and *Grk3*, were also observable in the ORs module. Studies revealed the impact of these genes on ORs regulation, as *Arrb2* inhibits the activation of ORs^36^ and *Grk3* has a role in ORs desensitization^37^.

For further investigation on the role of ORs in kidney fibrosis, another time-course UUO dataset was analyzed. LIMMA results determined 3176 DEGs (adjusted *p* value < 0.05), which were further used for the construction of a gene interaction network (Fig. 1f). Topological analysis of the constructed network revealed 44 significant modules, of which ORs-related module obtained the first rank (Fig. 1g). Overlaying the significantly expressed ORs between two datasets determined *Olfr433, Olfr129, Olfr1393, Olfr161*, and *Olfr622* as common ORs (Fig. 1h and Supplementary Fig. 1). These five common ORs were selected for in vivo expression analysis.

### Histopathological analysis of the UUO model validated the robustness of the constructed model

**Fig. 2:**
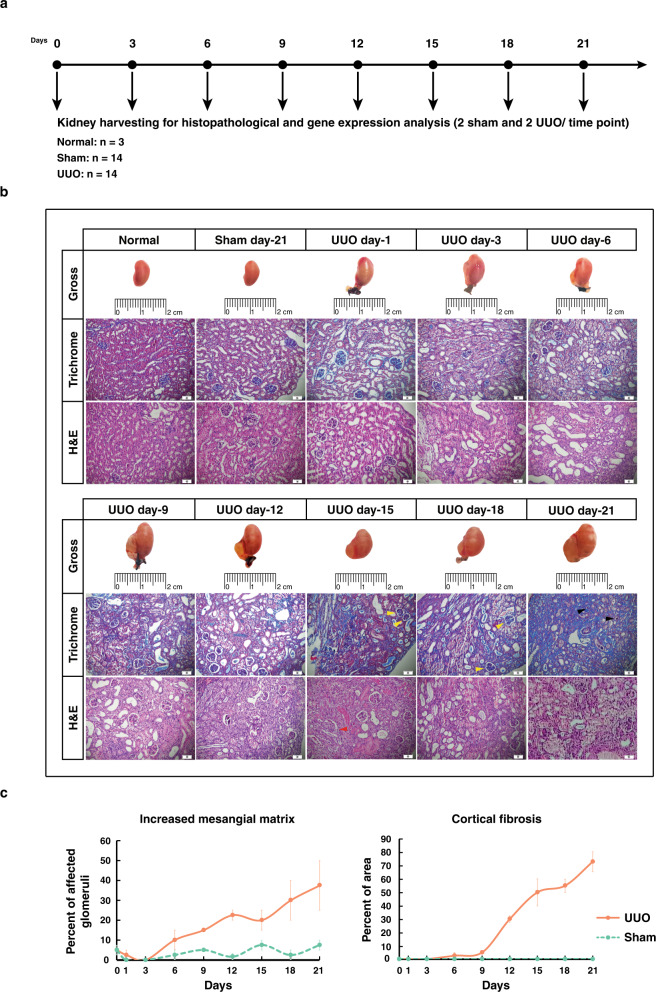
Histopathologic evaluation of unilateral ureteral obstruction (UUO) -operated mice over 21 days. The scheme of experimental design (**a**). Trichrome and hematoxylin and eosin (H&E) -stained renal sections in normal, sham (day-21), and UUO-operated mice at days-1, 3, 6, 9, 12, 15, 18, and 21 postsurgery. Yellow, red, and black arrows stand for increased mesangial matrix, mesangial cell proliferation, and diffuse glomerulosclerosis, respectively (**b**). The percentages of glomeruli with increased mesangial matrix (*p* value = 1.1451e-04) as well as cortical fibrosis (*p* value = 1.3868e-04) for sham and UUO-operated mice. Data are mean ± SEM (**c**). * Scale bars: 50 µm.

In order to experimentally evaluate the expression of selected ORs, a mouse model of UUO was developed. Both UUO and sham-operated mice were followed over 21 days (Fig. 2a). For validation of the quality of the constructed model, the histopathological analysis was performed. Assessment of the sections revealed significant diffused tubulointerstitial fibrosis along with glomerular injuries, increased mesangial matrix, and diffused glomerulosclerosis in UUO-operated mice compared to the sham group over 21 days of UUO treatment (*p* value < 0.05), all of which determined the robustness of constructed UUO model (Fig. 2b and c). Furthermore, to show that the developed animal model recapitulates what was reported in both studies by Wu et al., we assessed the expression of some of the genes by real-time quantitative polymerase chain reaction (RT-qPCR). In agreement with the results of study published in 2012^28^, *Hnf4a* was down-regulated and *Cebpb* was up-regulated in our model at day-12. Also, *Serpina3n* and *Tgfb1* that were shown to be up-regulated in a more recent study^29^, were increased in our expression analysis (Supplementary Table 1).

### Gene expression patterns demonstrated significant changes in ORs in the fibrotic kidney

As ORs belong to a highly conserved superfamily which most of them have a similar sequence, the specificity of designed primers was checked and validated by sanger sequencing (Supplementary Data 1).

**Fig. 3:**
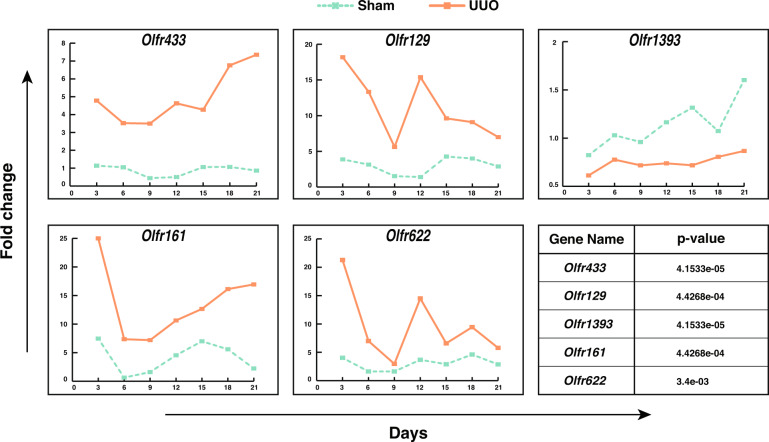
Expression level of common ORs between two datasets in a mouse model of UUO over 21 days. Statistical analysis (Friedman’s two-way ANOVA test) determined significant changes between UUO and sham groups for *Olfr433, Olfr129, Olfr1393, Olfr161*, and *Olfr622* (*p* value < 0.05).

The expression of *Olfr433, Olfr129, Olfr1393, Olfr161*, and *Olfr622* was evaluated over 21 days in a time-course manner in both UUO and sham-operated mice (Fig. 3 and Supplementary Data 2). Except for *Olfr1393*, other genes demonstrated upregulation over time in comparison to the sham group. To test whether these changes between the sham and UUO groups were significant during time, Friedman’s two-way ANOVA test was applied. The results of the analysis determined significant changes in patterns of expression in the UUO group compared to sham for all the genes (*p* value < 0.05). Considering the expression patterns over time revealed that *Olfr433, Olfr129, Olfr161*, and *Olfr622* had a sharp downregulation from day-3 to 6. Although *Olfr433* and *Olfr161* expression continued with a smoother augmenting response, *Olfr129* and *Olfr622* had a second sharp peak at day-12 followed by a reduction in the consecutive days. Furthermore, the expression of these four genes in the sham group over time revealed oscillatory patterns, which is an initial clue about the inherent rhythmic pattern of OR genes in the kidney.

### *Olfr433* is highly expressed by macrophages of the fibrotic kidney

**Fig. 4:**
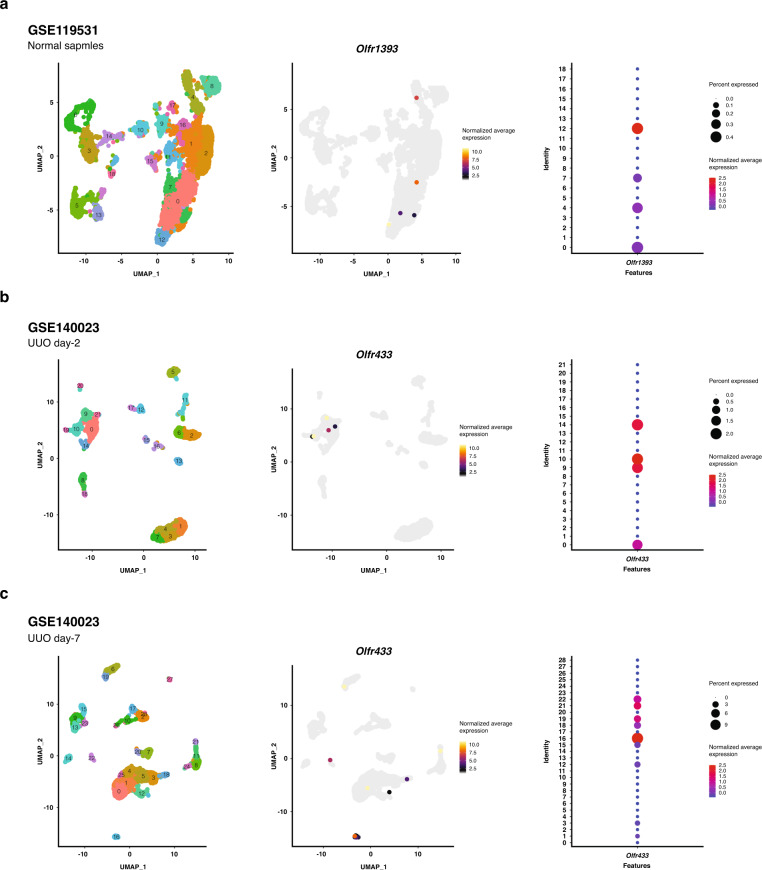
Localization of *Olfr1393* and *Olfr433* by analysis of two single-cell RNA-sequencing (scRNA-seq) datasets related to normal and UUO kidneys. Expression of *Olfr1393* in normal kidney (GSE119531) (**a**). Expression of *Olfr433* in UUO samples at day-2 (**b**) and 7 (**c**) postinjury (GSE140023). The left uniform manifold approximation and projection (UMAP) plots represent unsupervised clustering of the scRNA-seq datasets. The middle UMAP plots show the normalized average expression of ORs genes (yellow and grey color show high and low expression of genes). The dot plots indicate expression of *Olfr1393* and *Olfr433* across identified clusters (the circle size denotes percent of cells expressing OR genes and red and blue colors indicate high and low expression of OR genes, respectively).

To get insight into the kidney cell types expressing the selected ORs, publicly available scRNA-seq datasets of a normal kidney^38^ and a mouse model of UUO at day-2 and 7 postinjury^39^ were reanalyzed. After data quality control and removing unwanted cells, findings demonstrated expression of *Olfr1393* by proximal tubular (third segment) cells of the normal kidney. Consistent with our RT-qPCR results, no expression of *Olfr1393* was detected in the UUO samples. On the other hand, the expression of *Olfr433* was identified in both UUO day-2 and 7 postinjury. Our analysis showed that in day-2, *Olfr433* is mainly expressed by macrophages and to some extent proliferating proximal tubules. Likewise, in day-7 postinjury, *Olfr433* was shown to be predominantly expressed by macrophages (Fig. 4).

## Discussion

In order to acquire a holistic view of molecular mechanisms of kidney fibrosis, two time-course microarray datasets were reanalyzed. Gene interaction map evaluation determined modular structures of densely connected ORs in both networks. In the next step, to validate in silico results, the common ORs between two datasets were selected for further in vivo analysis in the mouse model of UUO. Additionally, the localization of ORs was inspected by analysis of kidney scRNA-seq datasets.

Although the relationship between some of the ORs and the normal physiology of the kidney has been determined in recent years^15,22,23^, few studies have focused on the role of ORs in pathological states of the kidney. The study by Shepard et al. focused on the role of *Olfr1393* in diabetes^24^. Additionally, our previous bioinformatic analysis determined a significant change in the expression of ORs in the rat model of ischemia-reperfusion injury^40^. In this study, based on in silico and in vivo investigations, we identified five ORs which all of them were significantly related to the progression of kidney fibrosis. Analysis of scRNA-seq datasets determined that *Olfr1393* is expressed by proximal tubular cells of the normal kidney. Similar to RT-qPCR results, no expression of this gene was detected in the UUO samples. These findings are in line with the results of the study by Shepard et al., which reported expression of *Olfr1393* in the proximal tubular cells of normal kidney^23^. On the other hand, our findings showed that *Olfr433* is predominantly expressed by macrophages of the injured kidney and not in normal kidney. The presence of macrophages in the site of injury is correlated with severity of tubulointerstitial damages^41^. It is shown that macrophages are capable of activating fibrotic pathways through which uncontrolled wound-healing processes and tissue fibrosis are promoted^42^. Study by Feng et al. demonstrated that transforming growth factor-beta (TGF-β) signaling pathway is activated in M2 macrophages in the injured kidney^43^. Also, wingless-related integration site (Wnt) signaling pathway in renal macrophages promotes their polarization into M2 phenotype and progression of fibrosis^44^. Moreover, macrophages are a potential source of extracellular matrix components like collagen^45^, platelet-derived growth factor (PDGF)^46^, and matrix metalloproteinase-9 (MMP9)^47^. This data provides a clue that this receptor has a potential role in inflammatory response and generation of myofibroblasts and is suggestive of the critical role of ORs in the pathogenesis of renal failure. We also appreciate that the localization of these ORs must be validated by other experimental approaches. Activation-inhibition approaches are also necessitated to determine the ORs function in kidney fibrosis.

In this study, we reanalyzed two qualified time-course microarray datasets generated by Wu et al. In both studies, key RNA biomarkers and molecular mechanisms of obstructive nephropathy were introduced^28,29^. Time-course study designs are worth as they would better demonstrate the dynamism of cellular behavior and also reduce misinterpretations that may occur in single-point studies^48,49^. Unfortunately, due to cost and difficulty, such experiments are less considered by investigators and most of the gene expression studies are performed statically^50^. On the other hand, appropriate analysis of time-series data is of great importance and should be performed by suitable mathematical approaches^32^. Although both studies by Wu et al. were designed temporally, single time-point analysis prevented them from finding significant changes in the expression of OR genes over the course of the disease.

Beside analyzing microarray datasets, we developed a time-course in vivo model of renal obstruction and followed both sham and UUO samples over 21 days. Expression analysis of *Olfr433, Olfr129, Olfr1393, Olfr161*, and *Olfr622* determined differential expression patterns for these genes during 21 days. For distinguishing real changes from noisy fluctuations, we compared the patterns in control and treatment groups. We appreciate that the inclusion of two mice per group in each time point is not an ideal sample size. However, based on the criteria discussed in our previous study^48^, we believe that this gene expression analysis is reliable as we performed time-course measurements for both UUO and sham groups. Considering that the differences were consistently observed in all-time points for all the examined genes, the alterations in gene expression were interpreted as real signals. In addition, the magnitude of fold changes was fairly high and the differences reached a statistically significant threshold. Moreover, evaluations determined rhythmic patterns in ORs expression in sham groups somehow similar to the rhythmic patterns, which is not far from the oscillatory function of the kidney^51,52^. These findings are only an initial clue about the rhythmic expression patterns of OR genes and further investigations on the relation of ORs expression patterns with kidney function would be of interest.

Taken together, these in silico and in vivo investigations validate the expression changes of ORs during the progression of kidney fibrosis. The time-course evaluations determined rhythmic and robust patterns of ORs expression and highlighted the potential role of *Olfr433* in the progression of kidney fibrosis. However, future studies are required for further validation of these findings. This study is a good example of the potential capacity of systems biology unsupervised top-down strategy to unravel the neglected aspects of disease pathogenesis.

## Material and Methods

### Microarray datasets analysis

The GSE36496 microarray dataset deposited by Wu et al.^28^ was downloaded from the gene expression omnibus (GEO) database^53^. The quality of the data was assessed by PCA and hierarchical clustering using ggplot2^54^ and pheatmap^55^ packages of R software^31^, respectively. To determine significantly expressed genes in this time-course dataset, we applied LIMMA^30^, a package of R software. Using the multiple comparison method of LIMMA, the sham and UUO groups were compared with each other at different time points and DEGs were determined by False Discovery Rate <0.05 (Benjamini–Hochberg). The second dataset, GSE96571 deposited by Wu et al.^29^, was also analyzed similarly, and DEGs were determined according to the previously mentioned criteria.

### Network construction and topological analysis

The DEGs were used for the construction of a gene-interaction network using the Cluepedia plugin^56^ (version 1.5.5) of Cytoscape software^57^ (version 3.7.2). The interaction data for activation, binding, inhibition, and post-translational modification with a confidence rate of 0.8, was retrieved from the search tool for the retrieval of interacting genes/proteins (STRING) database^58^ (STRING-ACTION-SCORE_v10.0_10090_09.06.2015). Based on the clustering coefficient parameter, the densely connected sites of the network with a cutoff point of 4 were determined by molecular complex detection (MCODE) plugin^59^ (version 1.5.1) as structural modules. The gene-interaction network for GSE96571 microarray dataset was also constructed, and the structural modules were investigated using the same method.

### Animal model of unilateral ureteral obstruction

Male C57BL/6 mice aged 6–8 weeks were obtained from Pasteur Institute (Tehran, Iran). Animal use and care were according to the guide for the use and care of animals by National Institutes of Health. Also, this study was approved by the Iranian national committee for ethics in biomedical research (Approval ID: IR.MUI.MED.REC.1398.323). For anesthesia, Ketamine and Xylazine (Alfasan, Woerden, Netherland) were injected at the dose of 115 and 11.5 mg/kg intraperitoneally. During surgery, mice were kept on a 37.5 °C plate. After a mid-abdominal incision, the left ureter was isolated and then double ligated. Afterward, the incision was sutured. In the sham group, all the steps were performed except ligation of the left ureter. The kidneys were harvested 1, 3, 6, 9, 12, 15, 18, and 21 days after surgery. Two UUO and two sham-operated mice were allocated for each time point. Additionally, three untreated mice were used as normal controls. After sacrificing with cervical dislocation, the left kidney was harvested and coronal sections were prepared. The anterior part was kept in 3.7% formaldehyde in PBS for histopathological study and the posterior part was sustained in liquid nitrogen for RNA extraction. As the in vivo study was performed to confirm the in silico results, we tried to recapitulate our model with the findings of the two reanalyzed datasets. In this regard, the expression of two prominent genes from each study was evaluated by RT-qPCR (Supplementary Table 1).

### Histopathological evaluation

The formalin-fixed kidney tissues were paraffin-embedded and 5 µm sections were prepared. Hematoxylin and eosin (H&E), as well as Masson trichrome (MT) staining, were carried out and histopathological evaluations were performed in a blinded manner. The existence of diffused glomerulosclerosis and mesangial cell proliferation were assessed between two experimental groups in random cortical fields using a X40 objective. Moreover, the percentage of cortex area affected by fibrosis including interstitial cortical fibrosis, tubular loss with minimal fibrosis, and periglomerular fibrosis were determined with the estimation of about five percent. Also, increased mesangial matrix was assessed by calculating the percentage of affected glomeruli in MT-stained sections. Measurements were repeated twice for each section.

### Statistical analysis

To compare the significance of histopathological differences between sham and UUO groups, Friedman’s two-way ANOVA test was applied using the ‘friedman’ function of MATLAB software (MathWorks, 2020b).

### Real-time quantitative polymerase chain reaction

Total RNA of the lower part of the left kidney was extracted using RNX-plus (CinnaGen, Tehran, Iran) according to the manufacturer’s instruction. Afterward, the concentration of the extracted RNA was measured by Epoch microplate spectrophotometer (BioTek, Winooski, Vermont). Since ORs sequence contains only the exon coding region, to eliminate DNA contamination, the samples were treated with DNase I (Thermo Fisher, Waltham, Massachusetts). To validate this procedure, mock controls were also employed (Supplementary Fig. 2). Subsequently, random hexamer primered cDNA synthesis was done using the first-strand cDNA synthesis kit (YektaTajhiz, Tehran, Iran). Specific primers for *Olfr433, Olfr129, Olfr1393, Olfr161, Olfr622, Hnf4a, Cebpb, Serpina3n, Tgfb1, Hprt*, and *Tfrc* were designed using AlleleID software^60^ (version 6.2) (Supplementary Table 2). RT-qPCR was performed using RealQ Plus 2x Master Mix Green with high ROX^TM^ (Ampliqon, Odense, Denmark) by Rotor-gene 6000 cycler (Qiagen, Hilden, Germany). The expression of genes was normalized by considering *Hprt* and *Tfrc* as internal control genes. The results of RT-qPCR were analyzed using the Pfaffl method by relative expression software tool (REST) version 1^61^.

### Statistical analysis

To compare gene expression patterns in UUO and sham groups, Friedman’s two-way ANOVA test was applied using the ‘friedman’ function of MATLAB software (MathWorks, 2020b).

### Sequencing of polymerase chain reaction products

PCR was performed by T100™ Thermal Cycler (Bio-Rad, Hercules, California) using Taq DNA Polymerase 2x Master Mix RED (Ampliqon, Odense, Denmark). The PCR products were cloned into the PTZ57R vector using TA Cloning™ kit (Thermo Fisher, Waltham, Massachusetts). Cloned products were transformed into competent TOP10 *E. coli* by incubating on ice with a subsequent heat-shock at 37 °C. Transformed colonies were cultured on LB-Agar (Sigma-Aldrich, St. Louis, Missouri) plate treated with ampicillin followed by overnight incubation at 37 °C. Afterward, plasmid extraction from bacteria was performed using Solg™ Plasmid Mini-prep Kit (SolGent, Daejeon, South Korea). Sanger sequencing of samples was done using both forward and reverse universal M13 (−40) primers by Bioneer biotech company (Daejeon, South Korea) (Supplementary Data 1).

### Single-cell RNA-sequencing datasets analysis

The gene barcode matrix of each dataset was downloaded using accession numbers GSE119531^38^ and GSE140023^39^ from GEO database^53^ and used as raw data to work using Seurat (version 3) package^62^. All downstream analysis was performed in Seurat package and each dataset was analyzed separately. To preprocess the data, including removing bad quality cells, the instructions provided by reference papers were followed. Briefly, for Wu et al., 2019 study^38^, the cells that contained more than 200, less than 4000 identified genes, and also contained less than 5 percent of mitochondrial genome were kept. For Conway et al., 2020 study^39^, these parameters were set as minimum of 300 genes, maximum of 3000 genes, and mitochondrial genes as less than 50 percent (according to the main paper settings). Subsequently, the gene expression level of cells was log-normalized and scaled. Downstream analysis was limited to 2000 highly variable genes to uncover more detailed differences between captured cells. PCA was performed on the highly variable genes to reduce the dimensionality of the data and the first 20 principal components were selected for the clustering purpose. Louvain clustering algorithm was used several times to identify clusters at multiple different resolutions and the optimum resolution was obtained according to distribution of DEGs. Finally, the uniform manifold approximation and projection (UMAP) algorithm was used to visualize the clusters in two-dimensional space. The cell type annotation was performed based on the expression of lineage-specific markers retrieved from related articles^39,63–66^ (Supplementary Fig. 3).

## Supplementary information

Supplementary information

## Data Availability

The UUO datasets analyzed during this study are available from GEO database using accession numbers GSE36496 and GSE96571. The results of PCR products sequencing are available at figshare repository (10.6084/m9.figshare.12753749.v11). The expression value of ORs can be downloaded from figshare repository (10.6084/m9.figshare.16755340.v2). ScRNA-seq datasets of normal and UUO samples are available in GEO database with the accession numbers GSE119531 and GSE140023.

## Code availability

The source code for LIMMA and scRNA-seq datasets analysis are available on GitHub: https://github.com/alimotahharynia/ORs-article/tree/analysis-code.
