# Supplementary material for: Olfactory receptors contribute to progression of kidney fibrosis
Source: NPJ Syst Biol Appl. 2022 Feb 18;8:8. doi: 10.1038/s41540-022-00217-w (PMC8857310; doi:10.1038/s41540-022-00217-w)
Supplement: Supplementary file 1 — Supplementary information [file 41540_2022_217_MOESM1_ESM.pdf]

Supplementary Information

Olfactory receptors contribute to progression of kidney fibrosis

Ali Motahharynia[1], Shiva Moein[1*], Farnoush Kiyanpour[1], Kobra Moradzadeh[1], Moein Yaqubi[2], Yousof Gheisari[1, 3]

1. Regenerative Medicine Research Center, Isfahan University of Medical Sciences, Isfahan, Iran

2. Department of Neurology and Neurosurgery, Montreal Neurological Institute and Hospital, McGill University, Montreal, Canada

3. Department of Genetics and Molecular Biology, Isfahan University of Medical Sciences, Isfahan, Iran

* Corresponding Author:

Shiva Moein, Ph.D. Regenerative Medicine Research Center, Isfahan University of Medical Sciences, Isfahan, 8174673461, Iran Tel/Fax: +98-3136687087.

Email: shiva.moein@res.mui.ac.ir

ORCID: 0000-0002-6019-9504

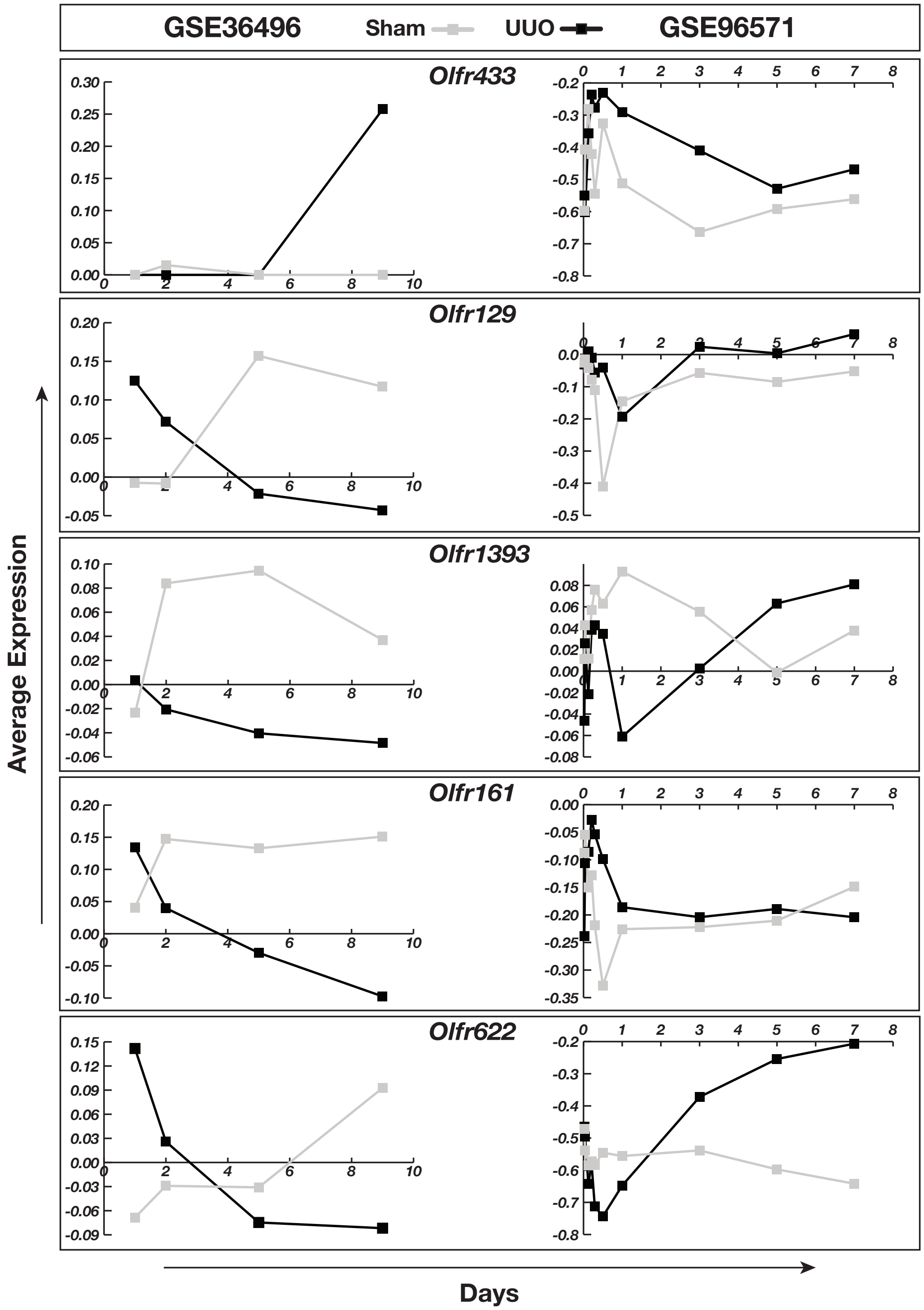

Supplementary Figure 1. Expression patterns of common ORs between GSE36496 and GSE96571 datasets. The expression patterns of common ORs (*Olfr433, Olfr129, Olfr1393, Olfr161,* and *Olfr622*) between GSE36496 and GSE96571 datasets. The right and left sides represent the expression patterns of ORs in GSE36496 and GSE96571 datasets, respectively.

Gene	Sham (FC)	UUO (FC)	Result
Cebpb	0.582	3.462 *	Up-regulation
Hnf4a	0.612	0.213 *	Down-regulation
Serpina3n	0.705 *	19.549 *	Up-regulation
Tgfb1	0.763 *	4.464 *	Up-regulation

Supplementary Table 1. Expression profile of selected genes confirms that our model recapitulates the findings of Wu et al. studies. Expression of each gene is normalized to two housekeeping genes (*Hprt* and *Tfrc*) and is shown as fold change (FC).

*** P-value < 0.05**

Supplementary Data 1. PCR products sequencing of *Olfr433, Olfr161, Olfr129, Olfr622,* and *Olfr1393*. The results were uploaded in figshare repository[1].

Supplementary Data 2. Expression value of *Olfr433, Olfr161, Olfr129, Olfr622,* and *Olfr1393* in mouse model of UUO over 21 days of treatment. The Cycle of threshold (Ct) alongside the amplification of each read were uploaded in figshare repository[2].

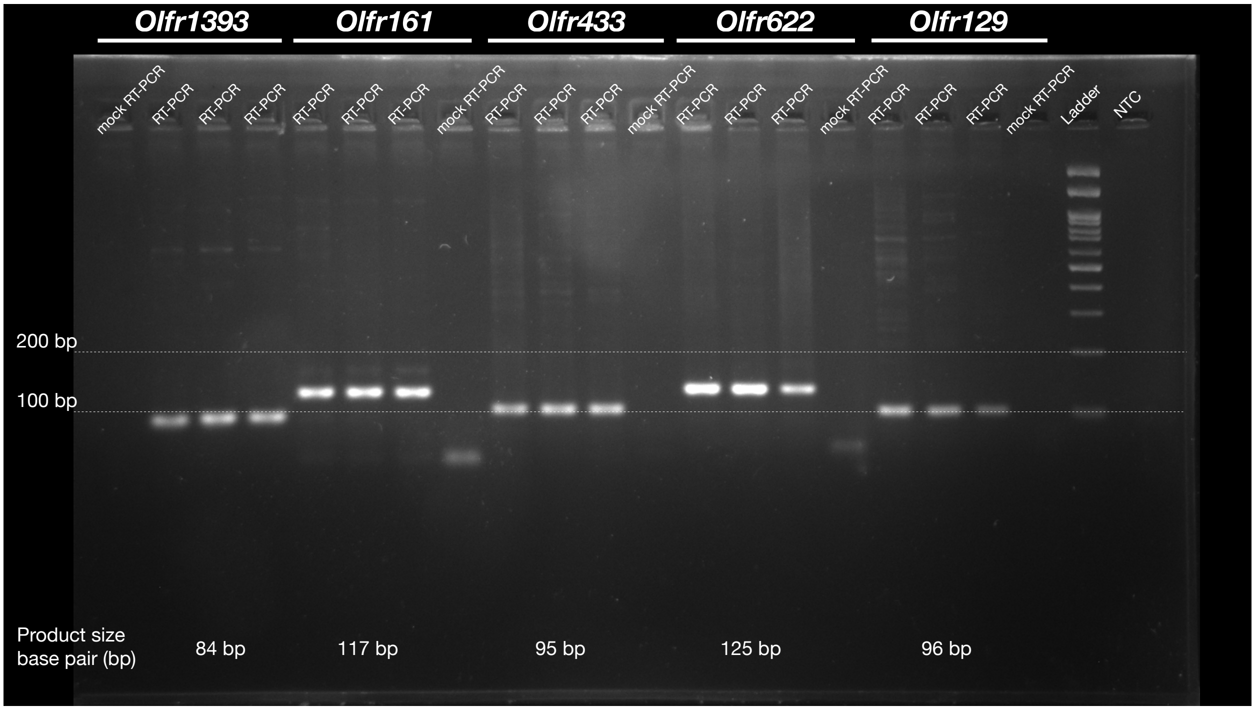

Supplementary Figure 2. The gel-electrophoresis of RT-qPCR products alongside their mock reactions. For each primer three different samples and mock reactions as well as non-template control (NTC) were loaded.

Gene	Gene ID	Forward primer	Reverse primer
Olfr433	258712	CTCCGACCTCTTGCCTTCC	CCTGAATCTGCCTGAACCAA
Olfr129	258324	GGTATAACTGAGTGCTGCCTACTG	CCCACGATTCATTCGGGTTGTAT
Olfr1393	258463	CGTCCACTGCACTATACT	GTTCAAGAAGCCTCCTACC
Olfr161	258859	GTCTTGGGAAACCTGCTCATCAT	AGTGGTGGAGGAGAAGCATACAT
Olfr622	259087	CCTGGCTTGGAGAATGTTCACTGT	ATAGGCTGGTGGAGACTTGGCTC
Cebpb	12608	CAACCTGGAGACGCAGCACAAG	GCTTGAACAAGTTCCGCAGGGT
Hnf4a	15378	ACTCCTCTCCTGCTATGCCA	GGGATGGTGACTCCTACCCT
Serpina3n	20716	CAACCCTGAACATCGGGAGT	TGCAGTCTACAGAGCTGAAACC
Tgfb1	21803	ATTCCTGGCGTTACCTTG	GCTGATCCCGTTGATTTC
Hprt	15452	CGTCGTGATTAGCGATGATG	AGTCTTTCAGTCCTGTCCATAA
Tfrc	22042	TGCATTGCGGACTGTAGAG	CCCACCAAACAAGTTAGAGAAT

Supplementary Table 2. The sequence of primers.

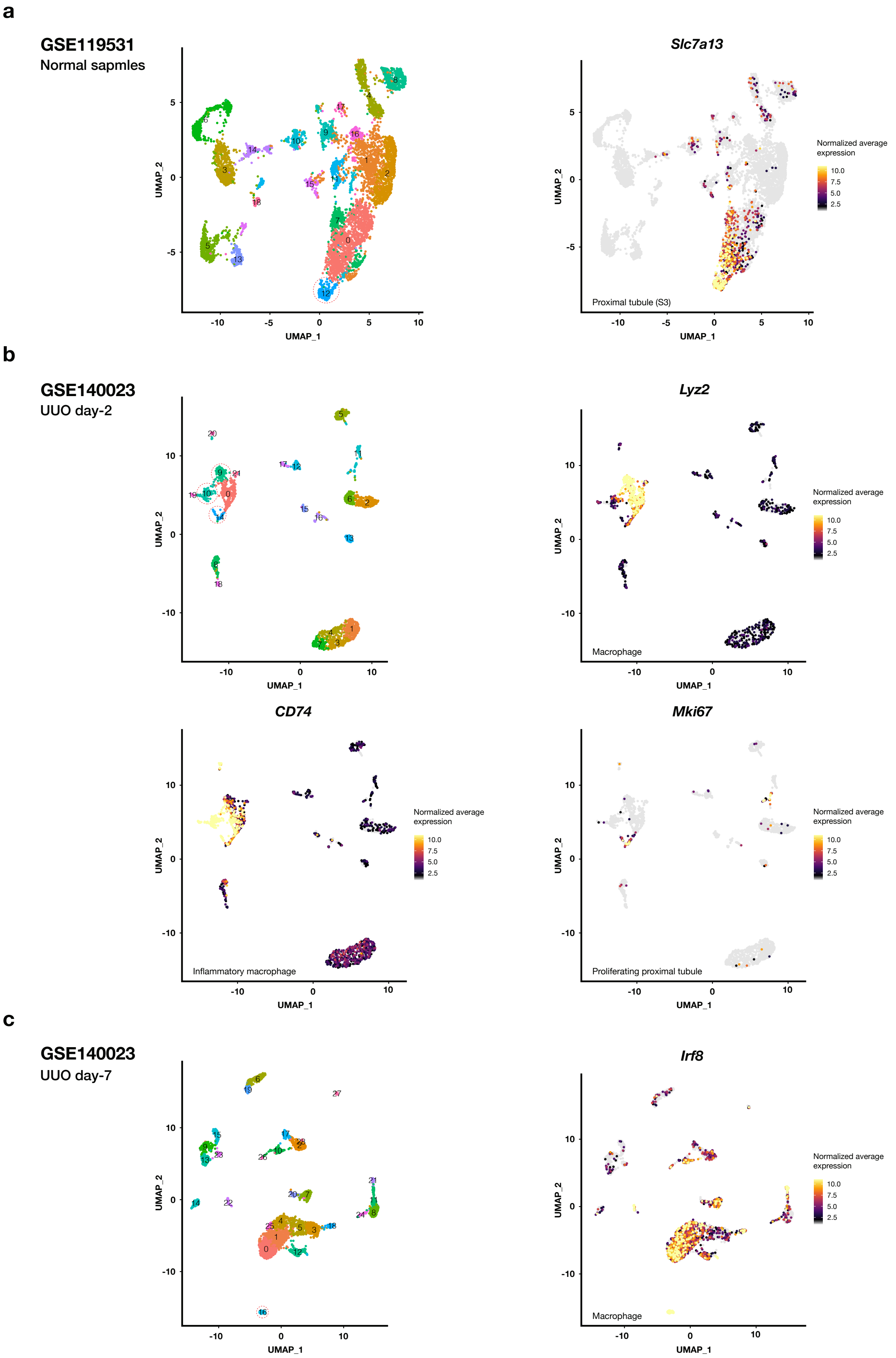
a
GSE119531
Normal sapmles
UMAP_1
UMAP_2
Slc7a13
Normalized average expression
Proximal tubule (S3)
b
GSE140023
UUO day-2
Lyz2
Macrophage
CD74
Inflammatory macrophage
Mki67
Proliferating proximal tubule
c
GSE140023
UUO day-7
Irf8
Macrophage

Supplementary Figure 3. The expression of selected marker genes related to normal and UUO kidneys. Expression of the proximal tubule third segment (S3) cell marker in normal kidney cells (GSE119531) along with its uniform manifold approximation and projection (UMAP) plot (a). Expression of macrophage, inflammatory macrophage, and proliferating proximal tubule cell marker in UUO day-2 and macrophage cell marker in UUO day-7 samples (GSE140023), with their related UMAP plots (b and c).

References

1. Motahharynia, A. *et al.* Supplementary Data 1: PCR products sequence. *Figshare* (2021) doi:https://doi.org/10.6084/m9.figshare.12753749.v11.
2. Motahharynia, A. *et al.* Supplementary Data 2: RT-qPCR results. *Figshare* (2021) doi:https://doi.org/10.6084/m9.figshare.16755340.v2.
